# The influence of “advancing” and “receding” colors on figure-ground perception under monocular and binocular viewing

**DOI:** 10.3758/s13414-024-02956-w

**Published:** 2024-09-30

**Authors:** Jaeseon Song, James M. Brown

**Affiliations:** 1 Department of Psychology, University of Georgia, Athens, GA 30602 USA; 2 Department of Psychological and Brain Sciences, University of Delaware, Newark, DE 19716 USA

**Keywords:** Figure-ground perception, Chromatic aberration, Magnocellular, Parvocellular

## Abstract

Research on figure-ground perception has consistently found that red images are more likely to be perceived as figure/nearer, yet the mechanisms behind this are not completely clear. The primary theories have pointed to optical chromatic aberrations or cortical mechanisms, such as the antagonistic interactions of the magno-/parvocellular (M/P) systems. Our study explored this color-biased figure-ground perception by examining the duration for which a region was perceived as figure under both binocular and monocular conditions, using all combinations of red, blue, green, and gray. In Experiment 1, we used figure-ground ambiguous Maltese crosses, composed of left- and right-tilting sectors of equal area. In Experiment 2, the crosses were figure-ground biased with size and orientation cues. Here, small sectors of cardinal orientations, likely perceived as figure, were contrasted with larger, obliquely oriented sectors, likely perceived as ground. Under monocular conditions, the results aligned with chromatic aberration predictions: red advanced and blue receded, regardless of size and orientation. However, under binocular conditions, the advancing effect of red continued, but the receding effect of blue was generally not observed. Notably, blue, along with red and green, was more frequently perceived as figure compared to gray. The results under binocular viewing are in line with the expectations of the antagonistic M/P system interactions theory, likely due to the collective input from both eyes, facilitating the anticipated effects. Our findings suggest that color-biased figure-ground perception may arise from the synergistic effect of antagonistic M/P system interactions and other optical and cortical mechanisms, together compensating for chromatic aberrations.

## Introduction

The human visual system has evolved to immediately organize a complex visual scene into figure (object or foreground) and ground (background). The figure is perceived as having a more definite shape and being in front of the ground, whereas the ground is seen as unformed, extending behind the figure. How does our brain readily segregate natural scenes we encounter every day? Psychologists first addressed this issue by examining the phenomenology of figure-ground perception along with stimulus factors influencing a region to be seen as figure, such as an area with smaller size (e.g., Graham, 1929), higher spatial (e.g., see Brown & Weisstein, 1988; Klymenko & Weisstein, 1986) and lower temporal (Klymenko & Weisstein, 1989a, b; Klymenko et al., 1989; Wong & Weisstein, 1984, 1985, 1987) frequency information, vertical and horizontal orientations (e.g., Oyama, 1960), greater contrast (Dresp-Langley & Reeves, 2014; Farne, 1977; Ichihara et al., 2007; Mount et al., 1956; Rohaly & Wilson, 1993), greater brightness (e.g., Camgoz et al., 2004; Egusa, 1983; Graham, 1929; Oyama, 1960; Oyama & Nanri, 1960), and hues (Brown & Greene, 2018; Brown & Plummer, 2020; Egusa, 1983; Graham, 1929; Guibal & Dresp, 2004; Humphrey, 1976; Luckiesh, 1918; Mount et al., 1956; Oyama, 1960; Plummer et al., 2018; Weisstein & Brannan, 1991). Particularly, many of the earlier works reported regions containing light of long wavelengths, such as red, tend to be perceived as figure or appear to be closer (i.e., advancing) in depth (Brown & Greene, 2018; Brown & Plummer, 2020; Egusa, 1983; Graham, 1929; Guibal & Dresp, 2004; Humphrey, 1976; Luckiesh, 1918; Oyama, 1960; Plummer et al., 2018; Weisstein & Brannan, 1991). There is no consensus on why red images stand out. It might be due to optical chromatic aberrations, known to contribute to blurred or defocused images projected onto the retina (Egusa, 1983; Kishto, 1965; Sundet, 1978; Vos, 2008; Winn et al., 1995), due to more complex brain mechanisms (Brown & Greene, 2018; Brown & Plummer, 2020; Faubert, 1994; Guibal & Dresp, 2004; Palmer & Brooks, 2008; Vecera et al., 2004; Weisstein & Brannan, 1991), or both.

Previous studies have shown that chromatic aberrations can act as cues for accommodation (for a review, see Del Águila‐Carrasco et al., 2020). To briefly review, there are two main types of chromatic aberration that could influence figure-ground perception: longitudinal chromatic aberration (LCA) and transverse (i.e., lateral) chromatic aberration (TCA). LCA refers to the variation in focal length for different light wavelengths, causing each color to have a different focal plane. For instance, blue light (short wavelength, S) converges before other colors, while red light (long wavelength, L) converges after green (middle wavelength, M). LCA alters the eye's focal power by nearly two diopters across the visible spectrum (Thibos et al., 1990). Notably, the chromatic defocus between the wavelengths of maximum sensitivity for S and M/L cones is approximately 1.5 D (Sprague et al., 2016; Thibos et al., 1992, p.3598), indicating that the visual impact of LCA could be more pronounced for blue than for red images.

Alongside LCA, which can occur in one eye, TCA also can play a role in figure-ground perception when viewing colored lights binocularly. Unlike LCA, TCA is characterized by lateral displacement of colors, typically with blue light being displaced more towards the center and red being displaced more towards the periphery (Thibos et al., 1990). TCA is known to contribute to chromostereopsis, a color stereoscopic effect where red tends to appear in front of blue in dark surrounds under binocular viewing (Hartridge, 1947; Sundet, 1978). This effect varies among individuals; some perceive blue as advancing, others see red receding, and some do not experience it at all (Simonet & Campbell, 1990b). Even for people who experience the phenomenon (i.e., red-in-front-of-blue), chromostereopsis occurrence depends on certain conditions, such as a dark surround (Dengler & Nitschke, 1993; Faubert, 1994; Vos, 2008), medium to high spatial frequency stimuli, as in stereograms (Faubert, 1994), and high illumination levels (Kishto, 1965; Simonet & Campbell, 1990a). Without these conditions, the phenomenon is less likely to occur. Additionally, chromostereopsis can be influenced or even negated by the Stiles-Crawford (SC) effect of the first kind, which describes how light entering through different parts of the pupil varies in effectiveness. Given the temporal position of the fovea and the nasal location of the pupil center (Yang et al., 2002), this off-axis position of the fovea, in conjunction with the SC effect, can interact with chromostereopsis caused by TCA. The interactions make chromostereopsis less perceivable in everyday life (Vos, 2008).

Although LCA and TCA can trigger accommodation, potentially influencing color-biased figure-ground perception, their overall impact seems limited. For instance, equiluminant colors do not typically induce accommodation, indicating a more subtle influence of these aberrations on visual perception (Switkes et al., 1990; Wolfe & Owens, 1981). Currently, there are no studies specifically investigating how figure-ground perception with colored stimuli is affected by LCA and TCA. Our approach to assessing their impact involves comparing how often red, green, or blue colors are perceived as figure versus ground under both monocular and binocular viewing conditions. This comparison was essential as binocular viewing is influenced by both LCA and TCA, while monocular viewing is only affected by LCA. It is yet to be determined whether these aberrations conform to their theoretical predictions under both viewing conditions, whether binocular viewing exhibits stronger effects due to the combination of LCA and TCA, or if the actual outcomes deviate from the predictions based on the aberrations.

We hypothesized that the impacts of LCA and TCA on figure-ground perception might be minimal, potentially mitigated by other optical phenomena like the SC effect and various brain mechanisms. These processes might compensate for the different focal points of colors resulting from chromatic aberrations, effectively offsetting the focus disparities across the color spectrum to a degree. This hypothesis aligns with evidence showing our visual system's ability to maintain high-resolution images despite optical aberrations (Artal et al., 2004; Benedi-Garcia et al., 2021; Dow, 2002; Nasr & Tootell, 2018; Sawides et al., 2011; Vautin & Dow, 1985; Yoshioka et al., 1996). If this is so, it leads to the question: if visual processes can offset focus disparities across the color spectrum, why do many studies report an advancing effect for red? We propose this might be explained by dynamic antagonism between the magnocellular (M) and parvocellular (P) systems, the two primary retino-geniculo-cortical visual pathways. Based on Weisstein’s antagonistic M/P system figure-ground model, P activity encodes figure and M activity encodes background (Brown & Greene, 2018; Brown & Plummer, 2020; Weisstein et al., 1992). In areas where a boundary separates two regions, the perception of figure or ground is determined by the interaction between M and P activities both within and across these regions. The region with a relatively stronger P “figure signal” is perceived as figure, whereas the one with a stronger M “ground signal” is perceived as ground. Thus, enhancing a region’s figure signal or diminishing its ground signal can lead to it being perceived as figure (i.e., as more advancing).

Research has shown that red light suppresses M activity (Bedwell et al., 2008; Breitmeyer & Breier, 1994), particularly in on-center type IV M neurons of the monkey LGN (de Monasterio, 1978; Wiesel & Hubel, 1966), possibly on-center type III M ganglion cells (de Monasterio & Schein, 1980) and cortical M cells (Livingstone & Hubel, 1984). Additionally, short wavelength sensitive S-cones are reported to minimally influence M cells (Chatterjee & Callaway, 2002; Dacey, 2000; Sun et al., 2006a, b). Therefore, red and blue light might reduce the M-biased ground signal, enhancing the P-biased figure signal and potentially altering the perception of these colors as figures.

Supporting the antagonistic M/P system figure-ground theory and cell recording findings, optical imaging (Garg et al., 2019; Liu et al., 2020; Salzmann et al., 2012; Xiao et al., 2007), and functional magnetic resonance imaging (fMRI) (Nasr & Tootell, 2018) have shown a bias in V1 responses towards end-spectral colors, such as red and blue, as opposed to mid-spectral colors like green and yellow. In V2 and V3, neural responses are categorized: P-dominant thin-stripes exhibit strong responses to mid-spectral colors like yellow and green, while also responding to a wide range of colors, including end-spectral colors. In contrast, M-dominant thick stripes almost exclusively respond to mid-spectral colors, suggesting that P thin-stripes respond relatively more strongly to end-spectral colors. The reduced response to red and blue in the thick stripes may result from red's suppressive effect on, and minimal S-cone input to, the M system.

The antagonistic M/P system figure-ground model agrees with LCA and TCA regarding the advancing effect of red. However, it diverges in its prediction about blue, suggesting an advancing effect for blue relative to green, contrasting with LCA and TCA. This suggests the perceptual prominence of red, and to a lesser extent blue, may be more influenced by the interplay of M and P system activities than by chromatic aberrations. This implies that after initial processes which equalize focus disparities across colors, red and blue become more perceptually salient.

Our study also aimed to evaluate how the advancing effect of red in figure-ground perception interacts with the influence of traditional cues such as size and orientation, with smaller size and cardinal orientations (horizontal and vertical) often linked to figures (e.g., Egusa, 1983; Graham, 1929). Additionally, we explored how green and blue behave in this setting, particularly in relation to these size and orientation cues.

To test our hypotheses, we used Maltese cross patterns to examine how figure-ground perception would be affected by red, green, blue, and gray stimuli presented under monocular (Experiments 1a and 2a) and binocular (Experiments 1b and 2b) viewing conditions, without (Experiment 1) and with (Experiment 2) figure-ground biasing size and orientation cues. Our independent measure was how long alternating crosses in the patterns were perceived as figure. In Experiment 1 the cross was designed to be figure-ground ambiguous featuring equal-area left- and right-tilting sectors. In Experiment 2 the cross was designed to be figure-ground biased with its smaller, cardinal-oriented sectors expected to be perceived as figure more often than its larger, obliquely oriented sectors. Equiluminant colors were used in all experiments to minimize chromatic aberration effects on eye accommodation (Switkes et al., 1990; Wolfe & Owens, 1981). To foreshadow, the results from Experiment 1a were somewhat aligned with LCA and TCA predictions, but they were more consistent with the antagonistic M/P system interactions theory in Experiment 1b.

## Experiments 1a and 1b

Experiments 1a and 1b used an ambiguous Maltese cross pattern, consisting of differently colored left- and right-tilting crosses of equal area, under monocular (1a) and binocular (1b) conditions. We measured the duration that crosses of different colors (red, green, gray, blue) were perceived as figure. According to LCA and TCA, red should advance while blue recedes, with green and gray positioned in between. We theorized that if chromostereopsis stemming from TCA is a key factor in color-biased binocular figure-ground perception, then the receding blue as well as advancing red effects should be observed and would diminish under monocular viewing with only LCA’s influence. Conversely, if the interplay of the M and P systems primarily governs figure-ground perception, especially for equiluminant colors, blue might appear more advancing than green and gray.

## Experiment 1a

### Method

#### Participants

Thirty-six participants (33 female, mean age = 19.0 years, SD = 1.9) from the University of Georgia were recruited and trained for this study. This total included one graduate student and five undergraduate research assistants who received course credit and were naïve to the purpose of the experiment. The others were students at the University of Georgia, who received partial course credit in their undergraduate psychology course for their participation. We verified that this sample size would provide sufficient power to detect the main effects of chromatic colors (red, green, and blue) compared to an achromatic color, gray, by conducting a power analysis using the “Bias and Uncertainty Corrected Sample Size” (BUCSS) R package (Anderson et al., 2017, Version 1.2.1). BUCSS uses the prior study’s observed T or F value and sample size, rather than provided estimates of effect size, to generate necessary sample sizes for planned studies. Along with the sample size of 15 from Egusa (1983), we used the F values of the reported main effects of chromatic colors compared to gray on depth perception (*F*(2, 28) = 15.76, *p* < .01) as inputs for the BUCSS “ss.power.wa()” function. The power analysis indicated a minimum sample size of five, when the assumed alpha level was set to 0.05, the desired level of statistical power was set to 0.8, and the desired level of assurance for correcting publication bias and uncertainty was set to 0.8.

To compare data from Experiments 1a and 1b, we conducted a power analysis using the ss.power.spa() function based on the previous study (Egusa, 1983) indicated along with the sample size of 15 and the F values (*F*(2, 28) = 15.76, *p* < .01) as inputs, a minimum per-group sample size of 17 for the between-subjects effect, when the assumed alpha level was set to 0.05, the desired level of statistical power was set to 0.8, and the desired level of assurance for correcting publication bias and uncertainty was set to 0.8. Experiment 1a exceeded the minimal sample size of 17 to ensure that our study reaches the desired statistical power.

Participants had normal or corrected-to-normal vision in both eyes and normal color vision (confirmed by pseudoisochromatic plates). All research conducted was under the approval of the University of Georgia Institutional Review Board’s ethical guidelines for research involving human participants.

#### Stimuli and apparatus

**Fig. 1:**
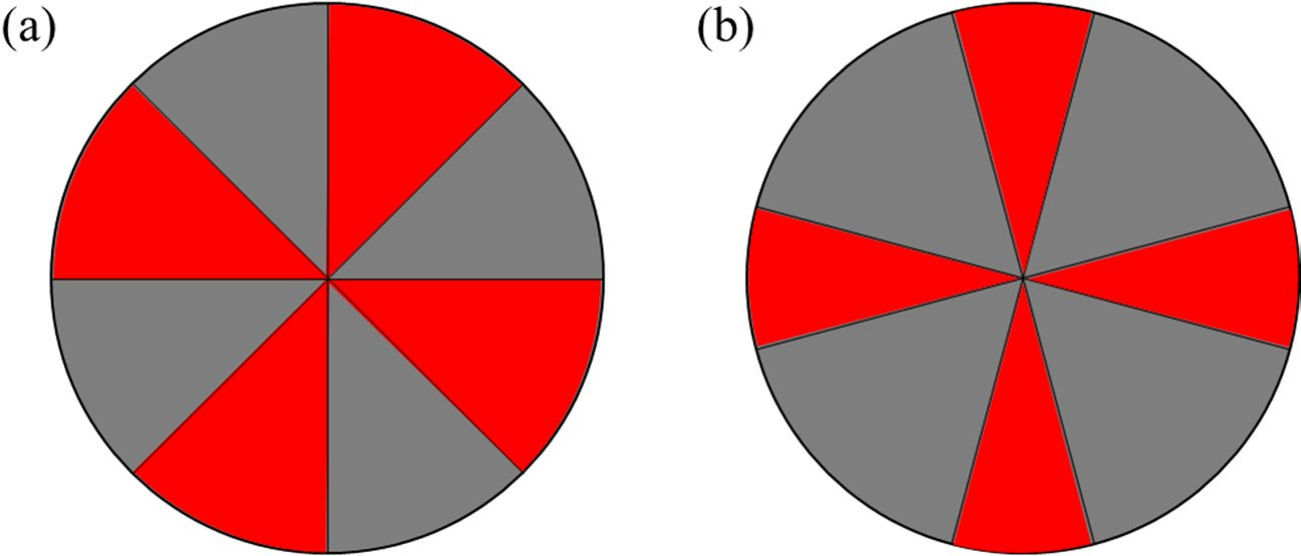
Examples of two types of patterns for the red and gray (Rd/Gy) pair used in our experiments. (**a**) An example of the ambiguous Maltese cross pattern from Experiment 1. This pattern comprises left- and right-tilting crosses of equal size, creating ambiguity in figure-ground perception. (**b**) An example of the Maltese cross pattern with figure-ground bias from Experiment 2, integrating size and orientation cues, featuring a smaller, cardinal oriented "plus-shaped cross," indicative of figures, and a larger, obliquely oriented "X-shaped cross," suggestive of grounds

Stimuli for all conditions had the same basic configuration. We used an ambiguous Maltese cross pattern (see Fig. 1a), which consisted of two regions – the "rightward tilted cross" and the "leftward tilted cross." Right and left refer to the location of the top of the cross with respect to the center of the pattern. Each of the two regions of the Maltese cross pattern consisted of four nonadjacent octants of a circular region. The diameter of the circular region was 4.6º of visual angle. The borders between the two regions were thin black lines and the horizontal, vertical, and two diagonal diameters of the circular region. The stimulus was presented against a black background. All sectors of the pattern were physically equiluminant, 15 cd/m^2^, and all colors were set to be physically equiluminant. The CIE xy coordinates were (x = 0.34, y = 0.34) for gray, (x = 0.65, y = 0.32) for red, (x = 0.22, y = 0.67) for green, and (x = 0.15, y = 0.08) for blue. Stimulus presentation and data collection were conducted using PsychoPy software (Version 1.90.3; Peirce, 2007) running on a PC equipped with a color LCD monitor operating at 60 Hz. For monocular viewing, participants wore a black opaque eye patch to cover their left eye. They were instructed to position their chin on a chin rest, maintaining a distance of 103 cm from the center of the monitor.

#### Design and procedure

Every possible color combination of red, blue, green, and gray was tested, resulting in six different color combinations (Rd/Gn, Rd/Bl, Gn/Bl, Rd/Gy, Gn/Gy, Gy/Bl). One single-color control condition (left-tilted Gy/right-tilted Gy) was also tested. A full display set was constructed with the controls to counterbalance the side (leftward tilted/rightward tilted) of the Maltese cross pattern. Participants were shown an individually randomized series of the full set of color combinations. They completed three practice trials, followed by 28 experimental trials. The dependent variable was the duration the left and right tilted crosses were perceived as figure in seconds.

Prior to the experiment, all participants underwent the three practice trials, using an orange/purple color combination, where they were familiarized with the stimuli and instructed how to do the task. No data from this session were used in the final analyses. After completing the training session, participants began their first experimental color combination.

During the experiment, an instruction screen was presented to participants, reminding them of their task and how to perform it. They were instructed to focus on the center of the stimulus and initiate each trial by pressing the space bar with their left hand. Participants were directed to press the “left” arrow key when the left-tilted cross appeared as the figure and the “right” arrow key for the right-tilted cross. If neither cross was perceived as the figure, they were not to press any key, thus establishing a three-alternative forced-choice setup. Participants had the option to revise their choice mid-trial if their perception of figure changed. Each trial lasted 40 s, and upon its completion, the total time for each of the three possible choices (left, right, or no figure) was summed up and recorded. Participants were asked to take additional short breaks if there was any afterimage remaining before continuing to the next trial. The experiment took approximately 42 min to complete. A one-way within-subjects ANOVA (color combination) with seven planned comparisons was performed on the mean durations each color was perceived as figure for the six color combinations (Rd/Gn, Rd/Bl, Gn/Bl, Rd/Gy, Gn/Gy, Gy/Bl) and one single-color control condition (left-tilted Gy/right-tilted Gy). We adjusted for Type 1 error inflation within each set of planned comparisons using the Benjamini-Hochberg correction with a base $\alpha$ value of 0.05 (Benjamini & Hochberg, 1995).

### Results

**Fig. 2:**
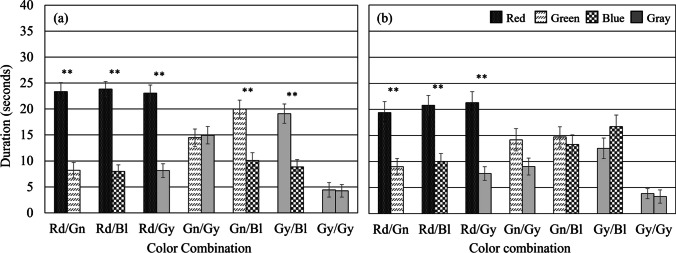
Durations the alternate crosses were perceived as figure in Experiment 1. Durations the alternate crosses were perceived as figure (**a**) under monocular viewing and (**b**) under binocular viewing, where the two crosses were figure-ground ambiguous, consisting of left- and right-tilting sectors of equal area. Error bars = $\pm$ *SEM*

The results are shown in Fig. 2a. A one-way within-subjects ANOVA was used to determine whether there are any statistically significant differences between the mean durations the alternate crosses were perceived as figure. Mauchly’s test indicated that the assumption of sphericity had been violated, *χ*^2^(90) = 381.977, *p* < .001, therefore degrees of freedom were corrected using Greenhouse-Geisser estimates of sphericity (*ε* = .349). The analysis with a Greenhouse-Geisser correction showed there was a significant effect of color combination, *F*(4.54, 158.92) = 24.2, *p* < .001, *η*^2^ = .409. Based on the predictions from chromatic aberration, five one-tailed planned comparisons (paired-sample t-tests) for the five color combinations Rd/Gn, Rd/Bl, Gn/Bl, Rd/Gy, Gy/Bl, and a two-tailed planned comparison for the Gn/Gy color combination were performed on the mean durations a color was perceived as figure. The five one-tailed planned comparisons showed that the durations were significantly longer for red crosses in Rd/Gn, *t*(35) = 5.32, *p* < .001, Rd/Bl, *t*(35) = 6.78, *p* < .001, and Rd/Gy conditions, *t*(35) = 5.89, *p* < .001, for green crosses in Gn/Bl condition, *t*(35) = 3.42, *p* = .001, and for gray crosses in Gy/Bl condition, *t*(35) = 3.46, *p* = .001. The five differences all remained significant after conducting Benjamini-Hochberg corrections. Two-tailed planned comparisons revealed the durations were not significantly different in the Gn/Gy condition, *t*(35) = -0.16, *p* = .872, nor between the mean durations for a left-tilted gray cross and a right-tilted gray cross in the Gy/Gy condition, *t*(35) = 0.12, *p* = .903.

## Experiment 1b

### Method

#### Participants

There were 32 undergraduate participants (26 female, mean age = 19.1 years, SD = 2.0), recruited for Experiment 1b using the same criteria as in Experiment 1a. Experiment 1b exceeded the minimal sample size of 17, indicated by the power analysis, to ensure that our study reached the desired statistical power.

#### Design and procedure

The design and procedure for Experiment 1b were the same as in Experiment 1a, except for the viewing condition with participants viewing displays binocularly. First, a one-way within-subjects ANOVA with seven planned comparisons was conducted. For our seven planned comparisons, we used Benjamini-Hochberg corrections to minimize the risk of Type I error. In addition, to directly compare figure-ground/depth effects with monocular (Experiment 1a) and binocular (Experiment 1b) viewing, the two data sets were combined, and a mixed two-way ANOVA was applied. When significant main effects and interactions were found in our mixed two-way ANOVA, Bonferroni-adjusted pairwise comparisons were run.

### Results

The results are shown in Fig. 2b. The one-way within-subjects ANOVA was used to determine whether there are any statistically significant differences between the mean durations the alternate crosses were perceived as figure. Mauchly’s test showed that the assumption of sphericity had been violated, χ^2^(90) = 390.33, *p* < .001, therefore degrees of freedom were corrected using Greenhouse-Geisser estimates of sphericity (*ε* = .293). The analysis with a Greenhouse-Geisser correction showed there was a significant effect of color combination, *F*(3.81, 118.20) = 12.09, *p* < .001, *η*^2^ = .281.

Based on the predictions from chromatic aberration, five one-tailed planned comparisons (paired-sample t-tests) for the five color combinations Rd/Gn, Rd/Bl, Rd/Gy, Gn/Bl, Gy/Bl, and a two-tailed planned comparison for the Gn/Gy color combination were performed on the mean durations a color cross was perceived as figure. One-tailed planned comparisons revealed that the durations were significantly longer for red crosses in Rd/Gn, *t*(31) = 3.21, *p* = .002, Rd/Bl, *t*(31) = 3.69, *p* < .001, and Rd/Gy conditions, *t*(31) = 4.62, *p* < .001. The three differences all remained significant after conducting Benjamini-Hochberg corrections. In this experiment, however, the durations were not significantly longer for green crosses in the Gn/Bl condition, *t*(31) = 0.45, *p =* .327, or for gray crosses in the Gy/Bl condition *t*(31) = -1.09, *p* = .142. Similar to Experiment 1a, two-tailed planned comparisons found no significant differences in the Gn/Gy condition, *t*(31) = 1.71, *p* = .097, nor between the mean durations for a left-tilted gray cross and a right-tilted gray cross in the Gy/Gy condition, *t*(31) = 0.37, *p* = .716. We additionally compared data from Experiment 1b to Experiment 1a. A mixed two-way ANOVA with viewing condition (monocular vs. binocular) as a between-subjects factor and color combination (Rd/Gn, Rd/Bl, Rd/Gy, Gn/Gy, Gn/Bl, Gy/Bl, Gy/Gy) as a within-subjects factor was applied to the combined data. Mauchly’s test indicated the assumption of sphericity had been violated, *χ*^2^(90) = 733.894, *p* < .001, therefore degrees of freedom were corrected using Greenhouse-Geisser estimates of sphericity (*ε* = .331). The analysis with a Greenhouse-Geisser correction showed the main effect of color combination was significant, *F*(4.31, 284.43) = 31.85, *p* < .001, *η*^2^ = .325, but not viewing condition, *F*(1, 66) = 1.13, *p* = .291, *η*^2^ = .017. The main effect was qualified by a significant interaction between color combination and viewing condition, *F*(4.31, 284.43) = 3.13, *p* = .013, *η*^2^ = .045. Bonferroni-adjusted comparisons indicated the duration was 5.28 s shorter for the green cross in Gn/Bl (*p* = .044), 5.91 s shorter for the gray cross in Gn/Gy (*p* = .015), 6.57 s shorter for the gray cross in Gy/Bl (*p* = .018), and 7.81 s longer for the blue cross in Gy/Bl (*p* = .003), under binocular viewing compared to monocular viewing. Levene’s test for equality of variances showed no significant difference between the variances of the monocular and binocular groups. Therefore, the equality of variances assumption is not violated.

### Discussion

The goal of Experiments 1a and 1b was to evaluate if color-biased figure-ground perception aligns with chromatic aberration predictions using an ambiguous pattern, under monocular and binocular viewing, respectively. In monocular vision, which would be solely influenced by LCA, we expected to observe red as advancing, green and gray as being in focus, and blue as receding. We anticipated that blue's receding effect, due to greater defocus than red, would be more pronounced. In binocular vision, both LCA and TCA would come into play. If conditions for chromostereopsis were met, it could extend the duration for which red is seen as the figure and blue as the ground. Conversely, if the dynamics of the M and P systems primarily drive color-biased figure-ground perception, we might not observe blue's receding effect, potentially leading to blue being more prominent than green and gray.

The results align with the predictions based on LCA under monocular viewing conditions (Experiment 1a). The duration red crosses were perceived as figure was longer than crosses of any other color. Green crosses were perceived as figure longer than blue crosses. Green and gray crosses were equally perceived as figure/ground. The duration blue crosses were perceived as figure was shorter than crosses of any other color. However, it is important to note that LCA alone cannot fully account for our results, since we observed a greater advancing effect of red in the Rd/Gn condition compared to the receding effect of blue in the Gn/Bl condition, whereas LCA predicts a greater defocus of blue than red. This implies that visual processes beyond LCA were involved in attempting to reduce focus disparities among different colors even in monocular vision.

In Experiment 1b, which involved binocular viewing, the advancing effect of red was observed, consistent with our findings from Experiment 1a under monocular viewing. However, interestingly, blue in the Gn/Bl and Gy/Bl conditions was more frequently perceived as the figure under binocular viewing compared to monocular viewing, aligning with the predictions made by the antagonistic M/P system interactions theory.

Moreover, although the differences were not statistically significant, there was a trend indicating that the green cross and even the blue cross tended to be perceived as the figure more often than the gray cross in Gn/Gy and Gy/Bl conditions, respectively. This tendency of chromatic colors, particularly blue, to be perceived as the figure more often than gray is inconsistent with the predictions from chromatic aberrations but is in line with predictions from the antagonistic M/P system interactions theory.

Nasr and Tootell (2018) revealed that P-dominant thin-stripes in V2 and V3 exhibit stronger responses to end-spectral colors compared to M-dominant thick stripes. According to the M/P system interactions theory, regions with relatively higher P activity tend to be perceived as the figure more frequently. Therefore, the observed advancing effect of blue, in contrast to green and gray, is consistent with both the fMRI findings and the M/P system interactions theory, suggesting that higher P activity, attributed to blue being an end-spectral color, accounts for this phenomenon.

The presence of TCA in binocular vision, which could have induced chromostereopsis, did not amplify the advancing effect of red or the receding effect of blue. In fact, the receding effect of blue vanished under binocular viewing, in contrast to monocular viewing. This inconsistency between our results and the predictions based on TCA and LCA suggests two key points. First, chromostereopsis may not readily occur unless specific stimulus requirements are met. Second, optical factors may not play a critical role in figure-ground perception with equiluminant colors during binocular vision; instead, brain mechanisms, such as the antagonism between the M and P systems, might complement and influence perception.

## Experiment 2

Experiment 2 investigated how color influences figure-ground perception under monocular (Experiment 2a) and binocular viewing (Experiment 2b) in the presence of the combination of two classical figure-ground cues, size and orientation. For size, a smaller region is more likely to be seen as figure (Graham, 1929; Oyama, 1960; Quinn & Bhatt, 2018). For orientation, cardinal orientations are more likely to be perceived as figure (Oyama, 1960; Quinn & Bhatt, 2018). In Experiments 2a and 2b, we modified the size and orientation of the Maltese crosses pattern (see Fig. 1b). The two crosses were figure-ground biased, consisting of a plus (+)-shaped cross of smaller sectors, presented at cardinal orientations, biased to be seen as figure, and an X-shaped cross of obliquely oriented larger sectors, biased to be seen as ground.

## Experiment 2a

In the monocular experiment (Experiment 1a), using an ambiguous configuration, the results aligned with LCA predictions: red crosses tended to be perceived as figures, and blue crosses as grounds, with green and gray crosses falling between these extremes. This suggests that when color and size and orientation cues align, as in a red plus-shaped cross, the perception of it as a figure could be enhanced. In contrast, in conditions like Gn/Gy or Gy/Gn, where LCA predicts similar perception, size and orientation cues alone might determine the figure-ground perception. When there is a conflict between color and size/orientation cues, it remains uncertain whether the influence of size and orientation would surpass that of color, or vice versa. This uncertainty highlighted an area for further investigation in understanding the relative strengths of these different perceptual cues in figure-ground perception.

### Method

#### Participants

There were 26 undergraduate participants (21 female, mean age = 18.5 years, SD = 1.0), recruited for Experiment 2a, using the same criteria as in Experiment 1. The power analysis indicated a minimum sample size of five (for the main effects of colors) and 22 (for the main effects of size and orientation) for a two-way within-subjects ANOVA, when the assumed alpha level was set to 0.05, the desired level of statistical power was set to 0.8, and the desired level of assurance for correcting publication bias and uncertainty was set to 0.8. Experiment 2a exceeded the minimal sample size of 22, the largest suggestion, to be conservative.

#### Stimuli and apparatus

Stimuli for Experiment 2a were created and presented the same way as in Experiment 1a, except for the Maltese cross pattern being modified (see Fig. 1b), consisting of two regions – the smaller, "plus-shaped cross," presented at cardinal orientations, and the larger "X-shaped cross," presented at oblique orientations. The plus-shaped cross was biased to be seen as figure, while the X-shaped cross was biased to be seen as ground. The diameter of the circular region was 4.6º of visual angle as in Experiment 1. The angle size of each smaller sector of the plus-shaped cross was 30º. The angle size of each larger sector of the X-shaped cross was 60º.

#### Design and procedure

Every possible figure-ground biased color combination of red, blue, green, and gray was tested, resulting in 12 different color combinations (Rd/Gn, Rd/Bl, Gn/Bl, Rd/Gy, Gn/Gy, Gy/Bl, Gn/Rd, Bl/Rd, Bl/Gn, Gy/Rd, Gy/Gn, Bl/Gy). One single-color control condition (Gy/Gy) was also tested, for a total of 13 figure-ground combinations. Participants viewed displays monocularly and were shown an individually randomized series of the full set of color combinations. They completed three practice trials, followed by 39 experimental trials. The dependent variable was the duration the plus- and X-shaped crosses were perceived as figure in seconds.

The experiment consisted of a 2 × 13 within-subjects measures ANOVA design, with size+orientation (plus-shaped vs. X-shaped crosses) and color combination (Rd/Gn, Rd/Bl, Gn/Bl, Rd/Gy, Gn/Gy, Gy/Bl, Gn/Rd, Bl/Rd, Bl/Gn, Gy/Rd, Gy/Gn, Bl/Gy, Gy/Gy) as factors. Six planned comparisons were conducted across five congruent-cues conditions (Rd/Gn, Rd/Bl, Gn/Bl, Rd/Gy, Gy/Bl) and the Gy/Gy condition, biased only by size and orientation. For the six planned comparisons, we used Benjamini-Hochberg corrections to minimize the risk of Type I error. For all other post hoc tests, Bonferroni-adjusted pairwise comparisons were run to explore significant main effects and interactions.

### Results

**Fig. 3:**
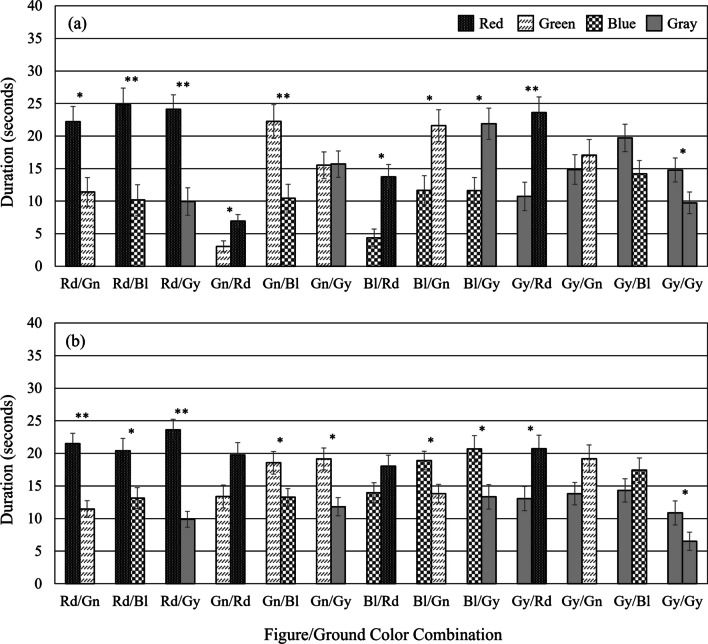
Durations the alternate crosses were perceived as figure in Experiment 2. Durations the alternate crosses were perceived as figure (**a**) under monocular viewing and (**b**) under binocular viewing, where the two crosses were figure-ground biased. In each figure-ground color combination, the bars on the left show the durations for Plus-shaped crosses, biased to be seen as figure, and the bars on the right show the X-shaped crosses, biased to be seen as ground. Error bars = $\pm$ SEM

The results are shown in Fig. 3a. A 2 × 13 within-subjects ANOVA (size+orientation × color combination) on duration perceived as figure was applied to duration data. Mauchly's test indicated that the assumption of sphericity had been violated, *χ*^2^(77) = 280.291, *p* < .001, therefore degrees of freedom were corrected using Greenhouse-Geisser estimates of sphericity (*ε* = .239). The analysis with a Greenhouse-Geisser correction showed the main effect of color combination, *F*(2.87, 71.74) = 55.83, *p* < .001, *η*^2^ = .691 was significant, but not the main effect of size/orientation, *F*(1, 25) = 0.56, *p* = .46, *η*^2^ = .022. The main effect was qualified by a significant interaction between color combination and size+orientation, *F*(3.48, 87.07) = 6.82, *p* < .001, *η*^2^ = .214.

Six one-tailed planned comparisons indicated that under monocular viewing, the duration for the Red-Plus cross was 10.82 s longer than the Green-X cross in Rd/Gn (*p* = .01), 14.64 s longer than the Blue-X in Rd/Bl (*p* = .002), and 14.19 s longer than the Gray-X in Rd/Gy (*p* = .001). The duration for the Green-Plus was 11.78 s longer than the Blue-X in Gn/Bl (*p* = .007), but not significantly longer for the Gray-Plus than the Blue-X in Gy/Bl. In Gy/Gy control condition, when only size and orientation were a factor, the duration was 5.05 s longer for the Gray-Plus than the Gray-X (*p* = .031). The differences all remained significant after conducting Benjamini-Hochberg corrections, except for the difference in Gy/Gy. Bonferroni-adjusted comparisons showed that even a red X-cross was seen significantly more often as figure in the Gn/Rd (*p* = .012), Bl/Rd (*p* = .001), and Gy/Rd (*p* = .007) conditions apparently having a stronger influence than the size and orientation cues. They also showed Bl/Gn (*p* = .035) and Bl/Gy (*p* = .022) were different, with green and gray X-crosses being seen more often as figure than blue plus-crosses, again contrary to size and orientation cues. There were no significant differences in the Gn/Gy, Gy/Bl, and Gy/Gn combinations.

## Experiment 2b

In Experiment 1b, under binocular viewing with color crosses and an ambiguous configuration, red crosses were seen as figure most often, consistent with the prediction from chromatic aberrations, but also blue and green crosses tended to be seen as figure more often than gray crosses, inconsistent with the predictions from chromatic aberrations. Therefore, here it was only predicted a Red-Plus cross should be perceived as figure more often than any of the Green-, Gray-, and Blue-X crosses. Again, a Gray-Plus cross should be seen as figure more often than a Gray-X cross.

### Method

#### Participants

There were 32 undergraduate participants (22 female, mean age = 19.1 years, SD = 1.0), recruited for Experiment 2b, using the same criteria as in previous experiments. Experiment 2b exceeded the minimal sample size of 22, indicated by the power analysis, to ensure that our study reaches the desired statistical power.

#### Design and procedure

The design and procedure for Experiment 2b were the same as in Experiment 2a, except participants viewed displays binocularly. A 2 × 13 within-subjects ANOVA design was applied. Four planned comparisons (paired-sample t-tests) were conducted across three conditions (Rd/Gn, Rd/Bl, Rd/Gy), based on the results in Experiment 1b and the Gy/Gy condition, biased only by size and orientation. To directly compare figure-ground/depth effects with monocular (Experiment 2a) and binocular (Experiment 2b) viewing, the two data sets were combined. A mixed three-way ANOVA (color combination × size+orientation × viewing condition) was then applied. For the four planned comparisons, we used Benjamini-Hochberg corrections to minimize the risk of Type I error. For other post hoc tests, Bonferroni-adjusted pairwise comparisons were run to explore significant main effects and interactions.

### Results

The results are shown in Fig. 3b. A 2 × 13 within-subjects ANOVA (size+orientation × color combination) on duration perceived as figure was applied to duration data. Mauchly’s test indicated that the assumption of sphericity had been violated, *χ*^2^(77) = 394.319, *p* < .001, therefore degrees of freedom were corrected using Greenhouse-Geisser estimates of sphericity (*ε* = .240). The analysis with a Greenhouse-Geisser correction showed there was a significant main effect of color combination, *F*(2.88, 89.12) = 22.01, *p* < .001, *η*^2^ = .415, and a significant main effect of size+orientation, *F*(1, 31) = 7.897, *p* = .009, *η*^2^ = .203. These main effects were qualified by a significant interaction between color combination and size+orientation, *F*(5.1, 158.12) = 6.2, *p* < .001, *η*^2^ = .167.

Four one-tailed planned comparisons indicated that under binocular viewing, for the Red-Plus cross, the duration it was seen as figure was 10.05 s longer than the Green-X cross in Rd/Gn (*p* < .001), 7.26 s longer than the Blue-X in Rd/Bl (*p* = .013), and 13.74 s longer than the Gray-X in Rd/Gy (*p* < .001). Again, in the Gy/Gy control condition, where size and orientation were the only factors, the Gray-Plus was seen as figure 4.36 s longer than the Gray-X (*p* = .013). The four differences all remained significant after conducting Benjamini- Hochberg corrections. Bonferroni-adjusted comparisons showed that there was a significant difference in Gn/Bl (*p* = .048), Gn/Gy (*p* = .003), Bl/Gn (*p* = .039), Bl/Gy (*p* = .049), and Gy/Rd (*p* = .04), but not in Gn/Rd, Bl/Rd, Gy/Gn, and Gy/Bl.

We additionally compared data from Experiment 2b to Experiment 2a. A 2 × 2 × 13 mixed three-way ANOVA with viewing condition (monocular vs. binocular) as a between-subjects factor and size+orientation (Plus vs. X) and color combination (Rd/Gn, Rd/Bl, Gn/Bl, Rd/Gy, Gn/Gy, Gy/Bl, Gn/Rd, Bl/Rd, Bl/Gn, Gy/Rd, Gy/Gn, Bl/Gy, Gy/Gy) as two within-subjects factors was applied to the combined data. Mauchly’s test indicated the assumption of sphericity had been violated, χ^2^(77) = 524.792, p < .001, therefore degrees of freedom were corrected using Greenhouse-Geisser estimates of sphericity (ε = .296). The analysis with a Greenhouse-Geisser correction revealed significant main effects of color combination, *F*(3.56, 199.15) = 48.13, *p* < .001, *η*^2^ = .462, and of size+orientation, *F*(1, 56) = 5.13, *p* = .027, *η*^2^ = .084, but not viewing condition, *F*(1, 56) = 0.81, *p* = .372, *η*^2^ = .014. There were significant interactions between color combination and viewing condition, *F*(3.56, 199.15) = 32.57, *p* < .001, *η*^2^ = .368, color combination and size+orientation, *F*(4.82, 270.04) = 10.66, *p* < .001, *η*^2^ = .16, and among all three factors, *F*(4.82, 270.04) = 3.1, *p* = .011, *η*^2^ = .053.

Bonferroni-adjusted comparisons indicated both the Green-Plus and the Red-X in Gn/Rd were seen as figure 10.35 s (*p* < .001) and 12.82 s longer (*p* < .001), respectively, under binocular compared to monocular viewing. The Blue-Plus in Bl/Rd was seen as figure 9.61 s longer under binocular viewing (*p* < .001). The Blue-Plus in Bl/Gn was seen as figure 7.23 s longer under binocular viewing (*p* = .007), while the Green-X was seen as figure 7.8 s longer under monocular viewing (*p* = .006). The Blue Plus in Bl/Gy was seen as figure 9.05 s longer under binocular viewing (*p* = .003), while the Gray-X was seen as figure 8.54 s longer under monocular viewing (*p* = .006). Levene’s tests for equality of variances showed significant differences for duration for each of the Green-Plus and the Red-X in Gn/Rd, and the Blue-Plus and the Green-X in Bl/Gn, indicating the assumption of underlying equal variances between the monocular and binocular groups could not be assumed. In other conditions, Levene’s tests revealed no differences between variances.

### Discussion

The purpose of Experiment 2 was to determine if color serves as a more potent figure-ground cue than traditional cues like size and orientation, under both monocular (Experiment 2a) and binocular (Experiment 2b) conditions. Predictions from LCA and TCA suggest red should appear closer, with green and gray in focus but slightly behind red, and blue noticeably further back. Due to the greater defocus of blue relative to red, blue's receding effect is expected to exceed red's advancing effect. Meanwhile, the antagonism between the M and P systems supports red's advancing effect but also predicts blue to advance more than green and gray.

On the other hand, the size and orientation cues in our modified Maltese cross pattern suggest that a plus-shaped cross (smaller, presented at cardinal orientations) would be more likely perceived as a figure, as opposed to an X-shaped cross (larger, presented at oblique orientations). Therefore, in certain conditions, the color cues (stemming from either chromatic aberration or the M/P system interactions) and size and orientation cues were aligned, creating congruence, while in others they were at odds, leading to incongruence in determining which regions were more frequently seen as figures.

In Experiment 2a, the tendency for red regions to be perceived as figures, observed in Experiment 1a, was again evident, regardless of size and orientation cues, aligning with predictions from LCA under monocular viewing. Particularly in congruent conditions (e.g., Red-Plus/Green-X, Green-Plus/Blue-X), results mirrored those of Experiment 1a, where LCA was the primary influence. However, in incongruent conditions (e.g., Green-Plus/Red-X, Blue-Plus/Green-X), while results largely supported LCA predictions, they also suggested additional factors influencing the perception of red and the relative defocus of blue. The duration red was seen as a figure was shorter in incongruent compared to congruent conditions, indicating a possible conflict between LCA and size and orientation cues.

Under binocular viewing in Experiment 2b, the influence of size and orientation cues was more pronounced. Red regions generally appeared as figures, but in incongruent conditions like Green-Plus/Red-X and Blue-Plus/Red-X, red X-crosses were not significantly more figure-like than green and blue plus-crosses, contrasting with monocular results. This suggests a conflict between size and orientation cues and chromatic aberrations under binocular viewing. Also, the plus-crosses being seen more often as figures in Gn/Bl and Bl/Gn conditions indicates a strong impact of these geometric cues. Even in conditions where chromatic aberrations predicted green over blue (Gn/Bl), the blue plus-crosses were seen as figures more often in the incongruent Bl/Gn, aligning with size/orientation cues. The consistent perception of colored regions (red, green, blue) as figures over gray in both Experiments 1b and 2b, regardless of their shape, suggests complex interactions between the M/P system interactions and geometric cues.

The combined analysis of Experiment 2a (monocular) and 2b (binocular) revealed intriguing differences, particularly with blue crosses. In the Bl/Rd condition, Blue-Plus crosses were perceived as figures more often under binocular than monocular viewing, while Red-X crosses showed consistent results across both. In the Bl/Gy condition, a reversal of trends was observed between monocular and binocular viewing for Blue-Plus and Gray-X crosses. These variations, particularly under binocular conditions, align more with the M/P system interactions theory than chromatic aberrations in determining figure-ground perception.

Similarly, the findings from Experiment 2, echoing Experiment 1, challenge the definitive role of chromatic aberrations in influencing color-biased figure-ground perception, especially in terms of red's advancing effect. Contrary to expectations based on chromatic aberrations, a stronger receding effect for blue compared to the advancing effect of red was hardly evident, particularly in binocular viewing. The reversal in perception trends under binocular conditions, particularly with blue more frequently appearing as the figure compared to green or gray, lends support to the antagonistic M/P system interactions theory. This observation in figure-ground perception indicates influences beyond chromatic aberrations, suggesting a significant role for the dynamic interaction between the M and P systems.

## General discussion

In this study, we explored whether the advancing effect of red in figure-ground perception is predominantly due to chromatic aberrations. Our findings corroborate previous research (Brown & Greene, 2018; Brown & Plummer, 2020; Egusa, 1983; Graham, 1929; Guibal & Dresp, 2004; Oyama, 1960; Weisstein & Brannan, 1991), indicating a bias towards perceiving red regions as figures. Under monocular conditions (Experiments 1a and 2a), it is assumed that LCA played a significant role in influencing figure-ground perception, thereby potentially reducing the impact of size and orientation cues. Participants appeared to rely more on chromatic aberrations rather than on size and orientation cues, especially when these cues conflicted. The results mostly aligned with LCA predictions, suggesting that red advances while green and gray are in focus, and blue recedes the most. However, the expectation of a greater defocus for blue compared to red, as per LCA, did not fully align with our observations, as red's figure effect was more pronounced than blue's ground effect.

In Experiments 1b and 2b, the influence of chromatic aberrations on figure-ground perception appeared diminished under binocular viewing. This could be potentially explained by the antagonistic M/P system interactions theory. The effects of red and blue light on M system activation in those image areas may be greater when viewing is binocular compared to viewing with just one eye possibly leading to a greater reduction in M system activity there. This, in turn, would increase P activity in those image areas biasing them to be perceived as figure. This explanation is especially relevant when considering the observed reversed effects of blue, in contrast to green and gray, under binocular as opposed to monocular conditions. Additionally, it was noted that not only red but also green and blue image areas were often seen as figures against gray, deviating from what chromatic aberration predictions would suggest. Furthermore, size and orientation cues became more significant, further indicating a diminished impact of chromatic aberrations.

Our results aligned with predictions from the antagonistic M/P system interactions theory under binocular viewing, despite using black lines as borders and a black background, fulfilling a key condition of chromostereopsis – a dark surround. Our results suggest that chromostereopsis is unlikely to be observed unless the specific conditions are met, such as medium to high spatial frequency components (Faubert, 1994), high levels of illumination (Kishto, 1965; Simonet & Campbell, 1990a), a dark surround (Dengler & Nitschke, 1993; Faubert, 1994; Vos, 2008), and binocular vision (Vos, 2008).

These findings imply that complex brain mechanisms, especially the dynamic interplay between the M and P systems, play a significant role in determining color-biased figure-ground perception in binocular vision, effectively mitigating the initial impacts of chromatic aberrations. This compensatory process might be aided by the interplay of various optical errors within the eyes, including additional off-axis effects of the pupil, a SC effect (Vos, 2008), and other forms of monochromatic and chromatic aberrations (Benedi-Garcia et al., 2021).

This study proposes a close interconnection between optical and perceptual factors in the advancing effect of red on figure-ground perception. It is essential to understand that neither optical nor perceptual factors alone can completely account for this effect. However, the antagonistic M/P system interactions are seen as a key contributor. This concept, elaborated in several works (Brown & Greene, 2018; Brown & Plummer, 2020; Plummer et al., 2018; Weisstein & Brannan, 1991), finds support in recent 7T high-resolution fMRI studies of the columnar organization in the human visual cortex (Nasr & Tootell, 2018; Tootell & Nasr, 2017).

Tootell and Nasr (2017) discovered segregated cortical columns in regions V2, V3, and V4 of the visual cortex, characterized by P-dominated thin stripes and M-dominated thick stripes. These stripes exhibited selective responses to stimuli known to elicit different reactions in the M and P systems of macaques, such as sensitivity to color versus luminance, binocular disparity, luminance contrast sensitivity, peak spatial frequency, and color/spatial interactions.

More recently, Nasr and Tootell (2018) observed a preference for achromatic (grayscale) and mid-spectral color (green) within the M-dominant thick stripes of V2 and V3. The absence of a response to red and blue in these thick stripes might be due to the suppressive effect of red on and the limited S-cone input to the M system. Consequently, our findings regarding the advancing effect of red in binocular vision might indicate that red amplifies the figure signal by reducing M activity. Similarly, the notably diminished receding effect of blue in binocular vision is consistent with a greater decrease in M activity for those image regions leading to an increased P figure signal there.

The significantly reduced receding effect of blue under binocular viewing in our experiments may also be a result of specific neuronal activities in a region of V2. Ts'o et al. (2001) identified a subset of cells in this region, selective for both color and disparity, in addition to other groups of cells, exclusively selective either for color or disparity. Without binocular stimulation, these dual-tuned cells, which may provide a counterbalance to chromatic aberrations, may remain inactive in monocular vision. Thus, this group of cells in V2 may contribute to mitigating the impact of chromatic aberrations, particularly serving as an accommodation cue in color images devoid of binocular disparity when viewed binocularly. This possibility needs to be further explored.

In conclusion, we propose that color-biased figure-ground perception arises from a complex interplay of optical and cortical mechanisms. This includes antagonistic interactions between the M and P systems, which appear to counteract chromatic aberrations, especially under binocular conditions. The process may also be influenced by various visual processes, including other optical aberrations and the activities of cells in V2 that are selective for both color and disparity. Our findings suggest an adaptation and calibration of our visual systems in response to the optical errors, aligning with insights from previous research (Artal et al., 2004; Benedi-Garcia et al., 2021; Dow, 2002; Nasr & Tootell, 2018; Sawides et al., 2011; Vautin & Dow, 1985; Yoshioka et al., 1996).

## Data Availability

Available upon request from the corresponding author.

## Code availability

Available upon request from the corresponding author.

## References

[CR1] Anderson, S. F., Kelley, K., & Maxwell, S. E. (2017). Sample-size planning for more accurate statistical power: A method adjusting sample effect sizes for publication bias and uncertainty. *Psychological Science,**28*(11), 1547–1562. 10.1177/0956797617723724 28902575 10.1177/0956797617723724

[CR2] Artal, P., Chen, L., Fernández, E. J., Singer, B., Manzanera, S., & Williams, D. R. (2004). Neural compensation for the eye’s optical aberrations. *Journal of Vision,**4*(4), 281–287. 10.1167/4.4.4 15134475 10.1167/4.4.4

[CR3] Bedwell, J. S., Brown, J. M., & Orem, D. M. (2008). The effect of a red background on location backward masking by structure. *Perception & Psychophysics,**70*(3), 503–507. 10.3758/pp.70.3.503 18459261 10.3758/pp.70.3.503

[CR4] Benedi-Garcia, C., Vinas, M., Dorronsoro, C., Burns, S. A., Peli, E., & Marcos, S. (2021). Vision is protected against blue defocus. *Scientific Reports,**11*(1), 1–9. 10.1038/s41598-020-79911-w 33432060 10.1038/s41598-020-79911-w PMC7801416

[CR5] Benjamini, Y., & Hochberg, Y. (1995). Controlling the false discovery rate: A practical and powerful approach to multiple testing. *Journal of the Royal Statistical Society: Series B (Methodological),**57*(1), 289–300. 10.1111/j.2517-6161.1995.tb02031.x

[CR6] Breitmeyer, B. G., & Breier, J. I. (1994). Effects of background color on reaction time to stimuli varying in size and contrast: Inferences about human M channels. *Vision Research,**34*(8), 1039–1045. 10.1016/0042-6989(94)90008-6 8160413 10.1016/0042-6989(94)90008-6

[CR7] Brown, J. M., & Greene, H. H. (2018). We’re going to study the mind! In J. M. Brown (Ed.), *Pioneer Visual Neuroscience: A Festschrift for Naomi Weisstein. *Routledge.

[CR8] Brown, J. M., & Plummer, R. (2020). When figure–ground segregation fails: Exploring antagonistic interactions in figure–ground perception. *Attention, Perception, & Psychophysics,**82*, 3618–3635. 10.3758/s13414-020-02097-w 10.3758/s13414-020-02097-w 32686064

[CR9] Brown, J. M., & Weisstein, N. (1988). A spatial frequency effect on perceived depth. *Perception & Psychophysics,**44*(2), 157–166. 10.3758/BF03208708 3405742 10.3758/bf03208708

[CR10] Camgoz, N., Yener, C., & Guvenc, D. (2004). Effects of hue, saturation, and brightness: Part 2: Attention. *Color Research and Application,**29*(1), 20–28. 10.1002/col.10214

[CR11] Chatterjee, S., & Callaway, E. M. (2002). S cone contributions to the magnocellular visual pathway in macaque monkey. *Neuron,**35*(6), 1135–1146. 10.1016/s0896-6273(02)00874-7 12354402 10.1016/s0896-6273(02)00874-7

[CR12] Dacey, D. M. (2000). Parallel pathways for spectral coding in primate retina. *Annual Review of Neuroscience,**23*(1), 743–775. 10.1146/annurev.neuro.23.1.743 10845080 10.1146/annurev.neuro.23.1.743

[CR13] de Monasterio, F. M. (1978). Properties of concentrically organized X and Y ganglion cells of macaque retina. *Journal of Neurophysiology,**41*(6), 1394–1417. 10.1152/jn.1978.41.6.1394 104012 10.1152/jn.1978.41.6.1394

[CR14] de Monasterio, F. M., & Schein, S. J. (1980). Protan-like spectral sensitivity of foveal Y ganglion cells of the retina of macaque monkeys. *The Journal of physiology,**299*(1), 385–396. 6770078 10.1113/jphysiol.1980.sp013131 PMC1279231

[CR15] Del Águila-Carrasco, A. J., Kruger, P. B., Lara, F., & López-Gil, N. (2020). Aberrations and accommodation. *Clinical and Experimental Optometry,**103*(1), 95–103. 31284325 10.1111/cxo.12938 PMC6911823

[CR16] Dengler, M., & Nitschke, W. (1993). Color stereopsis - a model for depth reversals based on border contrast. *Perception & Psychophysics,**53*(2), 150–156. 10.3758/Bf03211725 8433913 10.3758/bf03211725

[CR17] Dow, B. M. (2002). Orientation and color columns in monkey visual cortex. *Cereb Cortex,**12*(10), 1005–1015. 10.1093/cercor/12.10.1005 12217963 10.1093/cercor/12.10.1005

[CR18] Dresp-Langley, B., & Reeves, A. (2014). Effects of saturation and contrast polarity on the figure-ground organization of color on gray. *Frontiers in Psychology,**5*, 1136. 10.3389/fpsyg.2014.01136 25339931 10.3389/fpsyg.2014.01136 PMC4187611

[CR19] Egusa, H. (1983). Effects of brightness, hue, and saturation on perceived depth between adjacent regions in the visual field. *Perception,**12*(2), 167–175. 10.1068/p120167 6657423 10.1068/p120167

[CR20] Farne, M. (1977). Motion in depth induced by brightness changes in background. *Perception,**6*(3), 295–297. 10.1068/p060295 866086 10.1068/p060295

[CR21] Faubert, J. (1994). Seeing depth in colour: More than just what meets the eyes. *Vision Research,**34*(9), 1165–1186. 10.1016/0042-6989(94)90299-2 8184561 10.1016/0042-6989(94)90299-2

[CR22] Garg, A. K., Li, P. C., Rashid, M. S., & Callaway, E. M. (2019). Color and orientation are jointly coded and spatially organized in primate primary visual cortex. *Science,**364*(6447), 1275–1279. 10.1126/science.aaw5868 31249057 10.1126/science.aaw5868 PMC6689325

[CR23] Graham, C. H. (1929). Area, color, and brightness difference in a reversible configuration. *The Journal of General Psychology,**2*(4), 470–483. 10.1080/00221309.1929.9918085

[CR24] Guibal, C. R., & Dresp, B. (2004). Interaction of color and geometric cues in depth perception: When does “red” mean “near”? *Psychological research,**69*(1–2), 30–40. 10.1007/s00426-003-0167-0 14872343 10.1007/s00426-003-0167-0

[CR25] Hartridge, H. (1947). The visual perception of fine detail. *Philosophical Transactions of the Royal Society of London Series B, Biological Sciences,**232*(592), 519–671. http://www.jstor.org/stable/92320

[CR26] Humphrey, N. (1976). The colour currency of nature. In B. Mikellides (Ed.), *Colour for architecture* (pp. 95–98). Macmillan.

[CR27] Ichihara, S., Kitagawa, N., & Akutsu, H. (2007). Contrast and depth perception: Effects of texture contrast and area contrast. *Perception,**36*(5), 686–695. 10.1068/p5696 17624115 10.1068/p5696

[CR28] Kishto, B. N. (1965). The colour stereoscopic effect. *Vision Research,**5*(6), 313-IN314. 10.1016/0042-6989(65)90007-6 10.1016/0042-6989(65)90007-6 5905872

[CR29] Klymenko, V., & Weisstein, N. (1986). Spatial frequency differences can determine figure-ground organization. *Journal of Experimental Psychology: Human Perception and Performance,**12*(3), 324–330. 10.1037/0096-1523.12.3.324 2943860 10.1037//0096-1523.12.3.324

[CR30] Klymenko, V., & Weisstein, N. (1989). Figure and ground in space and time: 1. temporal response surfaces of perceptual organization. *Perception,**18*(5), 627–637. 10.1068/p180627 2602088 10.1068/p180627

[CR31] Klymenko, V., & Weisstein, N. (1989). Figure and ground in space and time: 2. frequency, velocity, and perceptual organization. *Perception,**18*(5), 639–648. 10.1068/p180639 2602089 10.1068/p180639

[CR32] Klymenko, V., Weisstein, N., Topolski, R., & Hsieh, C. H. (1989). Spatial and temporal frequency in figure-ground organization. *Perception & Psychophysics,**45*(5), 395–403. 10.3758/BF03210712 2726401 10.3758/bf03210712

[CR33] Liu, Y., Li, M., Zhang, X., Lu, Y. L., Gong, H. L., Yin, J. P., Chen, Z. Y., Qian, L. L., Yang, Y. P., Andolina, I. M., Shipp, S., Mcloughlin, N., Tang, S. M., & Wang, W. (2020). Hierarchical representation for chromatic processing across macaque V1, V2, and V4. *Neuron,**108*(3), 538-550.e5. 10.1016/j.neuron.2020.07.037 32853551 10.1016/j.neuron.2020.07.037

[CR34] Livingstone, M. S., & Hubel, D. H. (1984). Anatomy and physiology of a color system in the primate visual-cortex. *Journal of Neuroscience,**4*(1), 309–356. 10.1523/JNEUROSCI.04-01-00309.1984 6198495 10.1523/JNEUROSCI.04-01-00309.1984 PMC6564760

[CR35] Luckiesh, M. (1918). On " retiring " and "advancing " colors. *American Journal of Psychology,**29*, 182–186. 10.2307/1413561

[CR36] Mount, G. E., Case, H. W., Sanderson, J. W., & Brenner, R. (1956). Distance Judgment of Colored Objects. *Journal of General Psychology,**55*(2), 207–214. 10.1080/00221309.1956.9920312

[CR37] Nasr, S., & Tootell, R. B. H. (2018). Columnar organization of mid-spectral and end-spectral hue preferences in human visual cortex. *NeuroImage,**181*, 748–759. 10.1016/j.neuroimage.2018.07.053 30053514 10.1016/j.neuroimage.2018.07.053 PMC6263155

[CR38] Oyama, T. (1960). Figure-ground dominance as a function of sector angle, brightness, hue, and orientation. *Journal of Experimental Psychology,**60*(5), 299–305. 10.1037/h0041175 13731822 10.1037/h0041175

[CR39] Oyama, T., & Nanri, R. (1960). The effects of hue and brightness on the size perception. *Japanese Psychological Research,**2*(1), 13–20. 10.4992/psycholres1954.2.13

[CR40] Palmer, S. E., & Brooks, J. L. (2008). Edge-region grouping in figure-ground organization and depth perception. *Journal of Experimental Psychology: Human Perception and Performance,**34*, 1353–1371. 10.1037/a0012729 19045980 10.1037/a0012729 PMC2593880

[CR41] Peirce, J. W. (2007). PsychoPy–Psychophysics software in Python. *J Neurosci Methods,**162*(1–2), 8–13. 10.1016/j.jneumeth.2006.11.017 17254636 10.1016/j.jneumeth.2006.11.017 PMC2018741

[CR42] Plummer, R., Brown, J., & Song, J. (2018). Using artificial scotoma fading to explore antagonistic interactions in figure-ground perception. *Journal of Vision,**18*(10), 805–805. 10.1167/18.10.805

[CR43] Quinn, P. C., & Bhatt, R. S. (2018). Size and orientation cue figure-ground segregation in infants. *Visual cognition,**26*(7), 518–529. 10.1080/13506285.2018.1505794 31602175 10.1080/13506285.2018.1505794 PMC6786798

[CR44] Rohaly, A. M., & Wilson, H. R. (1993). The role of contrast in depth-perception. *Investigative ophthalmology & visual science,**34*(4), 1437–1437. <Go to ISI>://WOS:A1993KT89303635.

[CR45] Salzmann, M. F. V., Bartels, A., Logothetis, N. K., & Schuz, A. (2012). Color Blobs in Cortical Areas V1 and V2 of the New World Monkey Callithrix jacchus, Revealed by Non-Differential Optical Imaging. *Journal of Neuroscience,**32*(23), 7881–7894. 10.1523/Jneurosci.4832-11.2012 22674264 10.1523/JNEUROSCI.4832-11.2012 PMC6620961

[CR46] Sawides, L., de Gracia, P., Dorronsoro, C., Webster, M. A., & Marcos, S. (2011). Vision is adapted to the natural level of blur present in the retinal image. *PLoS one,**6*(11), 1–6. 10.1371/journal.pone.0027031 10.1371/journal.pone.0027031 PMC3206891 22073247

[CR47] Simonet, P., & Campbell, M. C. (1990). Effect of illuminance on the directions of chromostereopsis and transverse chromatic aberration observed with natural pupils. *Ophthalmic and Physiological Optics,**10*(3), 271–279. 10.1111/j.1475-1313.1990.tb00863.x 2216476

[CR48] Simonet, P., & Campbell, M. C. (1990). The optical transverse chromatic aberration on the fovea of the human eye. *Vision Research,**30*(2), 187–206. 10.1016/0042-6989(90)90035-j 2309454 10.1016/0042-6989(90)90035-j

[CR49] Sprague, W. W., Cooper, E. A., Reissier, S., Yellapragada, B., & Banks, M. S. (2016). The natural statistics of blur. *Journal of Vision,**16*(10), 23. 10.1167/16.10.23 27580043 10.1167/16.10.23 PMC5015925

[CR50] Sun, H., Smithson, H. E., Zaidi, Q., & Lee, B. B. (2006). Do magnocellular and parvocellular ganglion cells avoid short-wavelength cone input? *Visual Neuroscience,**23*(3–4), 441–446. 10.1017/S0952523806233042 16961978 10.1017/S0952523806233042 PMC2843149

[CR51] Sun, H., Smithson, H. E., Zaidi, Q., & Lee, B. B. (2006). Specificity of cone inputs to macaque retinal ganglion cells. *Journal of Neurophysiology,**95*(2), 837–849. 10.1152/jn.00714.2005 16424455 10.1152/jn.00714.2005 PMC2843159

[CR52] Sundet, J. M. (1978). Effects of colour on perceived depth: Review of experiments and evalutaion of theories. *Scandinavian Journal of Psychology,**19*(1), 133–143. 675178 10.1111/j.1467-9450.1978.tb00313.x

[CR53] Switkes, E., Bradley, A., & Schor, C. (1990). Readily visible changes in color contrast are insufficient to stimulate accommodation. *Vision Research,**30*(9), 1367–1376. 2219752 10.1016/0042-6989(90)90010-i

[CR54] Thibos, L. N., Bradley, A., Still, D. L., Zhang, X., & Howarth, P. A. (1990). Theory and measurement of ocular chromatic aberration. *Vision Research,**30*(1), 33–49. 10.1016/0042-6989(90)90126-6 2321365 10.1016/0042-6989(90)90126-6

[CR55] Thibos, L. N., Ye, M., Zhang, X., & Bradley, A. (1992). The chromatic eye: A new reduced-eye model of ocular chromatic aberration in humans. *Applied optics,**31*(19), 3594–3600. 10.1364/AO.31.003594 20725330 10.1364/AO.31.003594

[CR56] Tootell, R. B., & Nasr, S. (2017). Columnar segregation of magnocellular and parvocellular streams in human extrastriate cortex. *Journal of Neuroscience,**37*(33), 8014–8032. 10.1523/Jneurosci.0690-17.2017 28724749 10.1523/JNEUROSCI.0690-17.2017 PMC5559769

[CR57] Ts’o, D. Y., Roe, A. W., & Gilbert, C. D. (2001). A hierarchy of the functional organization for color, form and disparity in primate visual area V2. *Vision Res,**41*(10–11), 1333–1349. 10.1016/s0042-6989(01)00076-1 11322978 10.1016/s0042-6989(01)00076-1

[CR58] Vautin, R. G., & Dow, B. M. (1985). Color cell groups in foveal striate cortex of the behaving macaque. *J Neurophysiol,**54*(2), 273–292. 10.1152/jn.1985.54.2.273 4031988 10.1152/jn.1985.54.2.273

[CR59] Vecera, S. P., Flevaris, A. V., & Filapek, J. C. (2004). Exogenous spatial attention influences figure-ground assignment. *Psychological Science,**15*(1), 20–26. 10.1111/j.0963-7214.2004.01501004.x 14717827 10.1111/j.0963-7214.2004.01501004.x

[CR60] Vos, J. J. (2008). Depth in colour, a history of a chapter in physiologie optique amusante. *Clinical and Experimental Optometry,**91*(2), 139–147. 10.1111/j.1444-0938.2007.00212.x 18271777 10.1111/j.1444-0938.2007.00212.x

[CR61] Weisstein, N., & Brannan, J. R. (1991). A low spatial frequency, red sine wave grating will float in front of gratings with the same or similar spatial frequency but other chromaticities: M and P interactions in figure-ground perception. *Investigative Ophthalmology & Visual Science, Sarasota, Florida.,**32*(Suppl), 1274.

[CR62] Weisstein, N., Maguire, W., & Brannan, J. R. (1992). M and P pathways and the perception of figure and ground. In J. R. Brannan (Ed.), *Advances in psychology: Applications of parallel processing in vision* (vol. 86, pp. 137–166). Elsevier BV.

[CR63] Wiesel, T. N., & Hubel, D. H. (1966). Spatial and chromatic interactions in the lateral geniculate body of the rhesus monkey. *Journal of Neurophysiology,**29*(6), 1115–1156. 10.1152/jn.1966.29.6.1115 4961644 10.1152/jn.1966.29.6.1115

[CR64] Winn, B., Bradley, A., Strang, N. C., McGraw, P. V., & Thibos, L. N. (1995). Reversals of the colour-depth illusion explained by ocular chromatic aberration. *Vision Research,**35*(19), 2675–2684. 10.1016/0042-6989(95)00035-x 7483309 10.1016/0042-6989(95)00035-x

[CR65] Wolfe, J. M., & Owens, D. A. (1981). Is accommodation colorblind? Focusing chromatic contours. *Perception,**10*(1), 53–62. 7255083 10.1068/p100053

[CR66] Wong, E., & Weisstein, N. (1984). Flicker induces depth: Spatial and temporal factors in the perceptual segregation of flickering and nonflickering regions in depth. *Perception & Psychophysics,**35*(3), 229–236. 10.3758/bf03205936 6728621 10.3758/bf03205936

[CR67] Wong, E., & Weisstein, N. (1985). A new visual illusion: Flickering fields are localized in a depth plane behind nonflickering fields. *Perception,**14*(1), 13–17. 10.1068/p140013 4069931 10.1068/p140013

[CR68] Wong, E., & Weisstein, N. (1987). The effects of flicker on the perception of figure and ground. *Perception & Psychophysics,**41*(5), 440–448. 10.3758/bf03203037 3601626 10.3758/bf03203037

[CR69] Xiao, Y. P., Casti, A., Xiao, J., & Kaplan, E. (2007). Hue maps in primate striate cortex. *NeuroImage,**35*(2), 771–786. 10.1016/j.neuroimage.2006.11.059 17276087 10.1016/j.neuroimage.2006.11.059 PMC1892586

[CR70] Yang, Y., Thompson, K., & Burns, S. A. (2002). Pupil location under mesopic, photopic, and pharmacologically dilated conditions. *Investigative Ophthalmology & Visual Science,**43*(7), 2508–2512. https://www.ncbi.nlm.nih.gov/pubmed/12091457 12091457 PMC2989408

[CR71] Yoshioka, T., Dow, B. M., & Vautin, R. G. (1996). Neuronal mechanisms of color categorization in areas V1, V2 and V4 of macaque monkey visual cortex. *Behav Brain Res,**76*(1–2), 51–70. 10.1016/0166-4328(95)00183-2 8734043 10.1016/0166-4328(95)00183-2

